# Evaluation of BacLite *Rapid *MRSA, a rapid culture based screening test for the detection of ciprofloxacin and methicillin resistant *S. aureus *(MRSA) from screening swabs

**DOI:** 10.1186/1471-2180-6-83

**Published:** 2006-09-29

**Authors:** Gemma Johnson, Michael R Millar, Stuart Matthews, Margaret Skyrme, Peter Marsh, Emma Barringer, Stephen O'Hara, Mark Wilks

**Affiliations:** 1 Division of Infection, Barts and the London NHS Trust, London, UK; 2 Centre for Infectious Diseases, Institute of Cell and Molecular Sciences, Barts and the London School of Medicine and Dentistry, Queen Mary, University of London, UK; 3 Department of Microbiology, Salisbury District Hospital, Salisbury, UK; 4 Health Protection Agency South East, Southampton General Hospital, Southampton, UK; 5 Acolyte Biomedica, Porton Down Science Park, Salisbury, UK

## Abstract

**Background:**

Methicillin-resistant *Staphylococcus aureus *(MRSA) is a major nosocomial pathogen worldwide. The need for accurate and rapid screening methods to detect MRSA carriers has been clearly established. The performance of a novel assay, BacLite *Rapid *MRSA (Acolyte Biomedica, UK) for the rapid detection (5 h) and identification of hospital associated ciprofloxacin resistant strains of MRSA directly from nasal swab specimens was compared to that obtained by culture on Mannitol salt agar containing Oxacillin (MSAO) after 48 h incubation.

**Results:**

A total of 1382 nasal screening swabs were tested by multiple operators. The BacLite Rapid MRSA test detected 142 out of the 157 confirmed MRSA that were detected on MSAO giving a diagnostic sensitivity of 90.4, diagnostic specificity of 95.7% and a negative predictive value of 98.7%. Of the 15 false negatives obtained by the BacLite Rapid MRSA test, seven grew small amounts (< 10 colonies of MRSA) on the MSAO culture plate and five isolates were ciprofloxacin sensitive. However there were 13 confirmed BacLite MRSA positive samples, which were negative by the direct culture method, probably due to overgrowth on the MSAO plate. There were 53 false positive results obtained by the BacLite Rapid MRSA test at 5 h and 115 cases where MRSA colonies were tentatively identified on the MSAO plate when read at 48 h, and which subsequently proved not to be MRSA.

**Conclusion:**

The Baclite MRSA test is easy to use and provides a similar level of sensitivity to conventional culture for the detection of nasal carriage of MRSA with the advantage that the results are obtained much more rapidly.

## Background

The use of screening cultures to identify MRSA-colonised patients so that infection control measures can be implemented and prevent transmission to other patients, is well established [1]. Recent guidelines from the Society for Healthcare Epidemiology of America emphasize the importance of identifying reservoirs of nosocomial transmission by use of active screening methods [2]. However, traditional methods used to screen for MRSA rely on labour intensive and time consuming culture techniques which do not exclude MRSA for 48 h and may require a further 1–2 days to confirm positives [3]. The use of chromogenic media may reduce the time significantly, but there are few published studies on their efficacy [3]. During this time period, infection control measures, such as isolation, or cohorting of patients and prophylactic decontamination may be applied unnecessarily, or if not applied, unidentified MRSA-positive individuals may remain a hidden reservoir for cross infection. A rapid negative result should allow more effective use of hospital isolation resources, whilst a rapid positive result should help reduce the spread of the infection and MRSA infection rates

In recent years a variety of increasingly sophisticated DNA-based tests have been developed to detect MRSA carriage more rapidly [4,5]. Most of these assays are based on the detection of an *S. aureus*-specific sequence and the *mecA *gene, which encodes methicillin resistance, with a variety of methods to differentiate MRSA from methicillin resistant coagulase negative staphylococci which may also be present [6]. Despite the technical improvements in molecular based assays, their high cost and relatively high operator skill requirement remain obstacles to their widespread routine use.

This study describes the evaluation of the BacLite MRSA, a rapid culture based test which has been developed to detect ciprofloxacin resistant MRSA strains that have been associated with hospital acquired infections within the UK. The test measures Adenylate Kinase (AK) activity. Adenylate kinase (AK), is an essential 'house keeping' enzyme found inside all cells which regulates energy provision by catalysing the equilibrium reaction: ATP + AMP ↔ 2ADP. By supplying purified ADP *in vitro*, the reaction can be driven to generate up to 40,000 ATP molecules per minute. The amplified levels of ATP produced during a typical 5 minute reaction period can then be measured using the bioluminescent reaction of firefly luciferase. In this BacLite MRSA assay, AK detection is combined with selective broth enrichment, magnetic microparticle extraction and selective lysis of *S. aureus *to add target organism specificity. In the extraction step, paramagnetic micro-particles coupled with a mouse anti-*Staphylococcus aureus *monoclonal antibody are used to capture MRSA. The unbound fraction is removed by washing. Capture and washing occur as automated steps inside the automated wash module. In the lysis step, a reagent containing lysostaphin and ADP is added and the *S. aureus *in the sample lysed to release adenylate kinase (AK). The AK then catalyses conversion of ADP to ATP. Firefly luciferin and luciferase are added and light is emitted in the presence of ATP [7].

## Results

A total of 1382 nasal screening swabs were tested by multiple operators and the results are shown in the table. The BacLite Rapid MRSA test detected 142 out of the 157 confirmed MRSA that were detected on MSAO giving a diagnostic sensitivity of 90.4 and a diagnostic specificity of 95.7% (Table). This gives a negative predictive value of 98.7%. Of the 15 false negatives obtained by the BacLite Rapid MRSA test, seven grew small amounts (< 10 colonies of MRSA) on the MSA culture plate and five isolates were ciprofloxacin sensitive.

There were 53 false positive results obtained by the BacLite Rapid MRSA test of which approximately 70% were due to the presence of methicillin resistant coagulase negative staphylococci (MRCNS). There were 115 cases where suspect MRSA colonies were tentatively identified on the MSAO agar when read at 48 h, subcultured for further identification and subsequently proved not to be MRSA.

There were 13 confirmed BacLite MRSA positive samples which were negative by the reference method. In all these cases MRSA was isolated when the enriched BacLite broth was subcultured onto MSAO. In all cases MRSA was isolated, although in one case a non mannitol fermenting strain of MRSA was isolated There is no obvious explanation for the remaining 12 cases but in six of them the MSAO plates from the direct plating were recorded as being overgrown with commensal organisms which may have masked the presence of MRSA. The other cases may be due to the superior sensitivity of a broth enrichment method for the recovery of low numbers of organisms or have arisen simply by chance.

## Discussion

Laboratory screening for MRSA is a complex balance between turn around time, performance (sensitivity, specificity), ease of use, and cost. The majority of MRSA screening is carried out in clinical microbiology laboratories using plate based culture methods with or without prior broth enrichment. Broth based enrichment media enhance test sensitivity but adds an extra day to testing, and in any case the significance of the increased sensitivity, which is presumably due to increased recovery of low numbers of MRSA is not clear [3]. Direct plating onto solid media is therefore the most commonly used screening approach. There is no one solid medium that is clearly superior to any other for screening. MSAO is widely used as a screening medium both nationally and internationally and is one of two culture media (the other being ciprofloxacin containing Baird Parker medium) in the UK Health Protection Agency's national standard operating procedures, and was used in this study for comparison [3]. One major disadvantage of using MSAO is the growth of a large number of suspect colonies which require further testing and which are subsequently shown not to be MRSA. There is considerable individual variation between operators in the number of colonies which are tested. In this study, performed by several different operators, 115 out of 254 colonies which were sub cultured for further testing, were shown not to be MRSA, representing a substantial waste of resources. In comparison, there were 53 presumptive positives obtained by the BacLite *Rapid *MRSA which significantly reduces the number of follow up tests typically required by conventional methods.

The results of the study include 5 ciprofloxacin-sensitive (MIC = ≤ 6 mg/l) MRSA samples identified by the MSAO culture method which the BacLite assay did not detect, as it has been developed to detect the predominantly ciprofloxacin resistant MRSA strains that have been associated with hospital acquired infections within the UK. Ciprofloxacin containing media is widely used in the UK and although ciprofloxacin susceptible organisms will be missed the isolation rate with these media has been reported to be higher than other routinely used plate media like MSAO [3]. Of the 10 remaining false negative samples reported by the BacLite *Rapid *MRSA test, seven grew small amounts (< 10 colonies of MRSA) on the MSAO culture plate. It is possible that all culturable MRSA may have been removed from the swab when inoculating the MSAO plate prior to testing in the BacLite assay, or antibiotic treatment may have impacted on MRSA culturability increasing lag times or reducing organism growth rates.

Rapid identification or exclusion of MRSA colonization is increasingly seen as essential for the effective control of MRSA and other antibiotic resistant organisms in the hospital environment prompting the development of rapid tests for the detection of MRSA.

To reduce the time taken for this evaluation, wards were selected which had previously reported MRSA in the last 3 months, thus increasing the apparent prevalence of MRSA in the hospitals. This does not significantly affect the NPV but would have biased the positive predictive value[8]. For this reason, the latter is not shown. The high NPV of the BacLite Rapid MRSA (98.7%) test allows negative results to be confidently reported in 5 h. As negative samples make up the vast majority of MRSA screening tests this represents a significant benefit to laboratories. A useful feature of the assay is that it has been designed to retain a sample of the broth containing enriched MRSA from which to perform direct sensitivity tests and confirm presumptive positive results.

There were 13 confirmed BacLite MRSA positive samples which were negative by the reference culture method. Although they were classed as 'false positives' as the results were different from those of the reference method, they cannot be considered as false positives in the usual sense of the term, as a viable MRSA was isolated from the enriched Baclite broth. The better performance of the BacLite test in these samples was largely due to overgrowth of commensal organisms on the selective mannitol salt agar masking the presence of MRSA and, in one case the presence of a non mannitol fermenting strain of MRSA.

The material costs of the Baclite test are of the order of £5- 6 per test, which is higher than a culture based method, but much lower than commercial molecular based tests. The Baclite test was only evaluated in this study with nasal swabs, although similar results have been obtained from other body sites with screening swabs (Johnson Millar, Wilks, unpublished results). The Baclite test requires a relatively low level of expertise and can be performed by a trained laboratory assistant, whereas the skill mix required to operate a PCR system may not be readily available in the diagnostic laboratory.

A full cost benefit analysis was beyond the scope of this study. Such an analysis is extremely complex because it must take into account not only the costs and performance of the test in the laboratory, but arguably more important, how a rapid MRSA test is integrated into the workings of the laboratory and the hospital, which is difficult to quantify. In addition the benefits of a rapid test like the BacLite test or PCR, are dependent on factors which may not be under the laboratory control. For example the benefits of any time savings could be negated without an efficient specimen transport system in place, and an efficient method of reporting the results. An important consideration is the effect that a result obtained in about 5 h may have on the action taken by infection control staff. Specimens arriving for testing in the afternoon by the Baclite method may not be processed in time for infection control procedures to be implemented by the end of the day. Thus, action could not be taken until the following morning at the earliest. At present, because of the slowness of conventional culture methods and the shortening times of inpatient length of stay, action may not be taken on confirmed positive results, before the patient has been discharged. With a rapid test system, infection control facilities such as the number of isolation beds, may have to be increased to take into account the more rapid reporting of results.

## Conclusion

We describe here the first example of a rapid non molecular MRSA screening test.

The BacLite *Rapid *MRSA test is a sensitive and specific test for the detection of ciprofloxacin resistant MRSA nasal colonisation directly from a swab. The assay retains many of the advantages of traditional broth based methods but can provide an MRSA screening test result direct from a clinical swab within 5 hours. The test is easy to use and provides an alternative to conventional culture, or molecular approaches, for the detection of viable MRSA in routine clinical microbiology laboratories.

## Methods

### Clinical samples

From April to May 2005, the BacLite *Rapid *MRSA assay was evaluated by testing 1382 nasal screening swabs (cotton swabs with non-charcoal Amies transport medium, Medical Wire & Equipment, Wilts) from in-patients at 3 different hospitals within the United Kingdom. All swabs were taken as part of routine screening for MRSA colonisation according to local hospital infection control policies and anonymised according to local ethics committee requirements. To increase the sample positivity rate, wards were selected which had reported the presence of MRSA in the past 6 months. All specimens were transported and stored at room temperature and tested within 48 hours of sampling.

### Culture methods

All swabs samples were first spread on mannitol salt agar plates containing sodium chloride (5%) and oxacillin (4 mg/l) (MSAO agar, E & O Laboratories, Scotland) before processing by the Baclite method. After incubation at 37°C for 48 hours, the presence of mannitol-fermenting colonies with a staphylococcal morphology was recorded as a presumptive positive result.

MSAO plate culture presumptive positive isolates and the broth from BacLite presumptive positive samples were sub cultured onto horse blood agar, incubated overnight and colonies resembling *S. aureus *tested to confirm their identification by standard microbiology techniques (latex agglutination, deoxyribonuclease agar, tube coagulase). Methicillin resistance was confirmed by testing pure isolates for sensitivity to methicillin by UK standardized disc susceptibility methods [9].

### BacLite *Rapid *method

All specimens were processed according to the manufacturers instructions provided with the product. MSAO plates were inoculated first followed by the BacLite Rapid MRSA assay within 2 hours to avoid any bias due to processing delays. Positive and negative control strains (MRSA, MSSA) were included as procedural controls in each run. Swab samples were vortexed in the proprietary selective broth (containing ciprofloxacin (6 mg/l) for 2 × 5 sec and incubated at 37°C for 2.5 h. MRSA were captured and washed in the BacLite sample processor. The bound fraction was re-suspended in the selective broth and aliquots of each sample were placed into two adjacent wells of a 96 well assay plate (Corning Incorporated, NY). One well for each sample was used to determine a baseline signal (T0) in the BacLite reader. After a further incubation period of 2 hours at 37°C, the second well for each sample was processed in the same way (T2 reading). The result was determined by subtracting the T2 from the T0 reading and scored automatically as positive or negative according to a software embedded algorithm. Baclite assay-positive results were recorded as a presumptive positive and a sample of the incubated selective broth subcultured for confirmatory testing onto MSAO agar.

In addition, when a sample gave an MSAO presumptive positive reading (by colony morphology and presence of mannitol fermentation), but had given a BacLite negative reading, a ciprofloxacin susceptibility test was also carried out by standard methods [9].

### Quality controls

Reagent controls; 2 reagent controls were run with the assay, the non-inoculated broth control and the AK control. The broth control was assayed to detect contamination. The AK control was a monitor of bioluminescent reagent failure. An invalid result for either control invalidated the run.

Positive and negative controls were assayed with each run. The positive control (EMRSA 16, supplied by Acolyte Biomedica) monitored failure of the assay run. The negative control (Methicillin sensitive *S. aureus *strain NCTC 6571) was a procedural control and also monitored for any contamination of the assay. An incorrect result for either of these invalidated the run.

### Data analysis

Results from the BacLite *Rapid *MRSA assay were compared to those obtained after 48 h incubation of swabs inoculated onto MSAO agar. The total number of confirmed MRSA-positive samples obtained from MSAO plate was taken as the reference method in this study. Sensitivity, susceptibility, and negative predictive value (NPV) were calculated.

## Abbreviations

Adenylate Kinase (AK)

Adenosine triphospate ATP

Adenosine diphosphate ADP

Adenosine monophospahte AMP

Mannitol salt agar containing Oxacillin (MSAO)

Methicillin-resistant *Staphylococcus aureus *(MRSA)

Methicillin resistant coagulase-negative staphylococci (MRCNS)

Minimum inhibitory concentration (MIC)

National Collection of Type Cultures (NCTC)

Polymerase chain reaction (PCR)

## Competing interests

EB and SO'H are employees of Acolyte Biomedica Ltd who have developed the BacLite MRSA test under evaluation.

## Authors' contributions

MM, EB, SO' H and MW designed the study. GJ, SM, MS, PB and EB carried out the study. GJ, SM, MS PB SO'H and MW analysed the data. GJ, MM SO'H and MW wrote the manuscript. All authors read and approved the final manuscript.

## Figures and Tables

**Table 1:** Comparison of the BacLite *Rapid *MRSA test results with those obtained by the reference method

		BacLite MRSA positive	BacLite MRSA negative	Total
Reference method: MSAO culture and confirmatory tests	Positive	142	15	157
	Negative	53	1172	1225
	Total	195	1187	1382
